# Fine-mapping of a QTL and identification of candidate genes associated with the lateral branch angle of peanuts (*Arachis hypogaea* L.) on chromosome B05

**DOI:** 10.3389/fpls.2024.1476274

**Published:** 2024-10-03

**Authors:** Hongtao Deng, Xiukun Li, Shunli Cui, Li Li, Qinglin Meng, Yanxia Shang, Yingru Liu, Mingyu Hou, Lifeng Liu

**Affiliations:** ^1^ State Key Laboratory of North China Crop Improvement and Regulation/Key Laboratory for Crop Germplasm Resources of Hebei/North China Key Laboratory for Crop Germplasm Resources of Education Ministry/Hebei Agricultural University, Baoding, China; ^2^ School of Landscape and Ecological Engineering, Hebei University of Engineering, Handan, China

**Keywords:** peanuts, lateral branch angle, fine-mapping, F-box, real-time quantitative

## Abstract

Peanuts play a crucial role as an oil crop, serving not only as a primary source of edible oil but also offering ample protein and vitamins for human consumption. The lateral branch angle of peanuts is the angle between the main stem and the first pair of lateral branches, which is an important agronomic trait of peanuts, significantly impacts the peg penetration into the soil, plant growth, and pod yield. It is closely intertwined with planting density, cultivation techniques, and mechanized harvesting methods. Therefore, the lateral branch angle holds substantial importance in enhancing peanut yield and facilitating mechanization. In order to conduct in-depth research on the lateral branch angle of peanuts, this research is grounded in the QTL mapping findings, specifically focusing on the QTL *qGH* associated with the lateral branch angle of peanuts located on chromosome B05 (142610834-146688220). By using Jihua 5 and PZ42 for backcrossing, a BC1F2 population comprising 8000 individual plants was established. Molecular markers were then developed to screen the offspring plants, recombine individual plants, conduct fine mapping. he results showed that using the phenotype and genotype of 464 recombinant individual plants selected from 8000 offspring, narrow down the localization interval to 48kb, and designate it as *qLBA*. The gene *Arahy.C4FM6Y*, responsible for the F-Box protein, was identified within *qLBA* through screening. Real-time quantitative detection of *Arahy.C4FM6Y* was carried out using M130 and Jihua 5, revealing that the expression level of *Arahy.C4FM6Y* at the junction of the main stem and the first lateral branch of peanuts was lower in M130 compared to Jihua 5 during the growth period of the first lateral branch from 1 to 10 centimeters. Consequently, *Arahy.C4FM6Y* emerges as a gene that restrains the increase in the angle of the first lateral branch in peanuts. This investigation offers novel genetic reservoirs for peanut plant type breeding and furnishes a theoretical foundation for molecular marker-assisted peanut breeding.

## Introduction

Peanut is a significant economic and oilseed crop worldwide, playing a crucial role in meeting the demand for high-quality edible oil and protein. Increasing peanut yield and mechanized production are key objectives in peanut breeding. Peanuts are nutritionally rich, with a kernel fat content of approximately 50%, protein content of around 25%, and sugar content of about 10% (Pandey et al., 2012; Arya et al., 2016). Additionally, they contain various beneficial ingredients such as vitamins, minerals, essential amino acids, and unsaturated fatty acids (Kayam et al., 2017). Widely cultivated across continents like Asia, Africa, and the Americas, peanuts have an annual production of roughly 35.5 million tons. China holds a dominant position in the global peanut industry, serving as a significant production and consumption center and ranking among the world’s leaders in planting scale and yield (Gallavotti, 2013).

For crop yield, plant type plays a crucial role. Plant type encompasses the spatial morphology and distribution characteristics of roots, stems, leaves, branches, and flowers. In 1968, Donald introduced the concept of ‘idotype’ to describe the ‘ideal plant type’ for crops, emphasizing low competition among individuals and maximum biomass accumulation in grains (Donald, 1968). He suggested that the ideal wheat plant type should have an upright and short stem, a large spike, awns, and short leaves. This ideal plant type can significantly boost crop yield, with lateral branches being a key component that influences yield. Peanuts’ lateral branches originate from axillary buds on the main stem, drawing nutrients from it (Wang et al., 2018; Ward and Leyser, 2004). Effective branches, capable of bearing pods, are distinguished from ineffective branches. Peanut plant types are categorized into upright, semi spreading type and spreading type based on the angles of lateral branches and the ratio of the first pair’s length to the main stem’s height (plant type index) (Hammons et al., 2016). Creeping lateral branches, which grow close to the ground with slightly raised tops and a plant type index of about 2 or more, fall into the creeping type. The lateral branches of half-erection plants bend upwards from the base, while the upper part grows upright, with the upright part being greater in length than the curved part, resulting in a plant type index of approximately 1.5. On the other hand, the lateral branches of upright plants also bend upwards from the base, with the angle between the first pair of lateral branches and the main stem being less than 45°, leading to a plant type index of about 1.1-1.2. The main peanut varieties currently promoted in China are upright, featuring compact plants with pods primarily concentrated at the bottom, making them suitable for high-density cultivation and economically viable for small-scale farmers (Chen et al., 2016). The completion of genome sequencing in cultivated peanuts has ushered in a new era of functional genomics research, particularly in the realm of peanut plant types (Chen et al., 2019; Zhuang et al., 2019; Bertioli et al., 2019). Research utilizing SSR and transposon markers has led to the construction of a genetic map, identifying two major QTLs controlling branching angle on 2 linkage groups (Shirasawa et al., 2012). Another study pinpointed the collateral angle in a 4.08 Mb interval at the end of chromosome B05 using the RIL population (Li et al., 2019a). Furthermore, through the use of synthetic tetraploid and fleur11 to create chromosome fragment replacement lines and BSA analysis, researchers successfully mapped the genes governing the relative traits of lateral spreading and erect growth to the end of A08 linkage group and B05 chromosome, with the candidate gene being bunch1 (Fonceka et al., 2012).

Erect type peanuts have some drawbacks as the early flowers on the base of lateral branches are close to the ground, allowing pegs to form pods, while the later flowers higher up cannot. In comparison, semi spreading type and spreading type peanuts can conserve seed usage due to wider lateral branch angles and increased coverage (Zhou et al., 2016). Their lower lateral branches enable pegs to efficiently enter the soil, boosting per plant yield and facilitating mechanized harvesting. Additionally, post-harvest, they can be easily air-dried on site, reducing the risk of Aspergillus flavus contamination. The lateral branch angle of peanuts plays a vital role in yield, disease resistance, and mechanized harvesting, yet the genetic and molecular mechanisms behind this trait remain unclear.

The offspring plants produced by backcrossing PZ42 and Jihua 5 undergo recombination within the QTL interval. Reduce QTL interval through fine mapping and identify major genes related to peanut lateral branch angle. This study aims to uncover the genetics of peanut lateral branch angle and identify potential key genes, ultimately advancing molecular breeding and enhancing peanut germplasm resources.

## Method

### Construction of secondary mapping population

**Figure 1:**
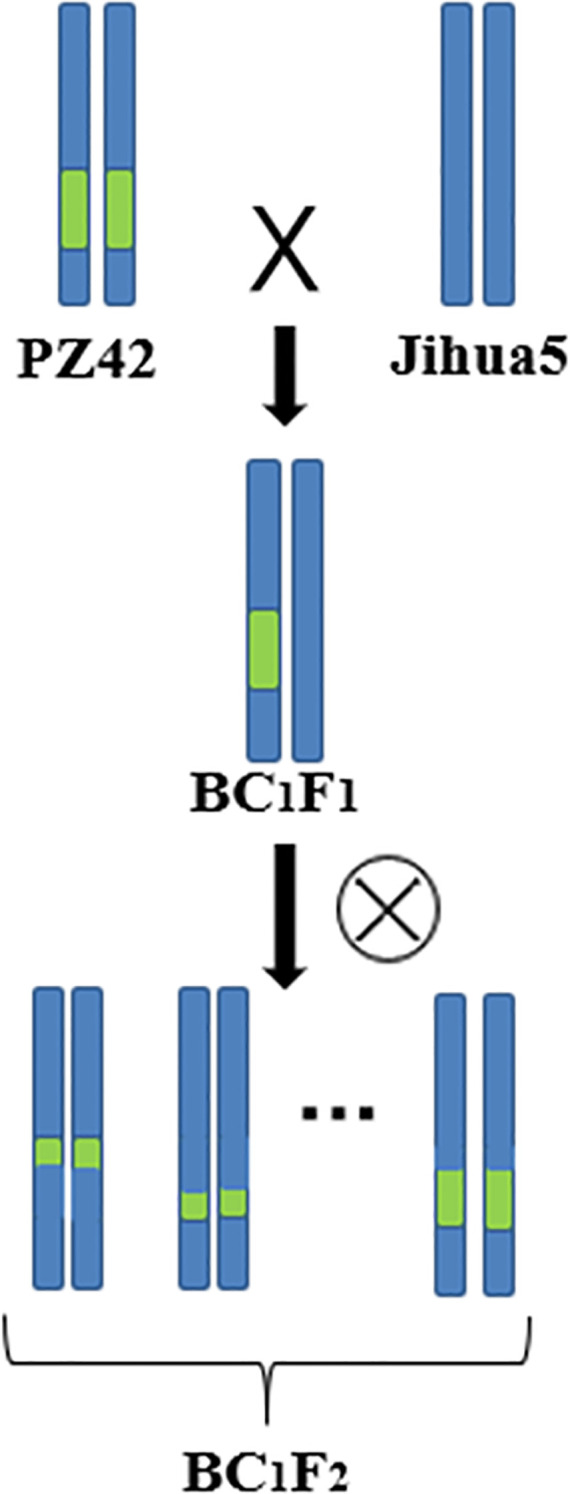
Secondary population construction.

**Figure 2:**
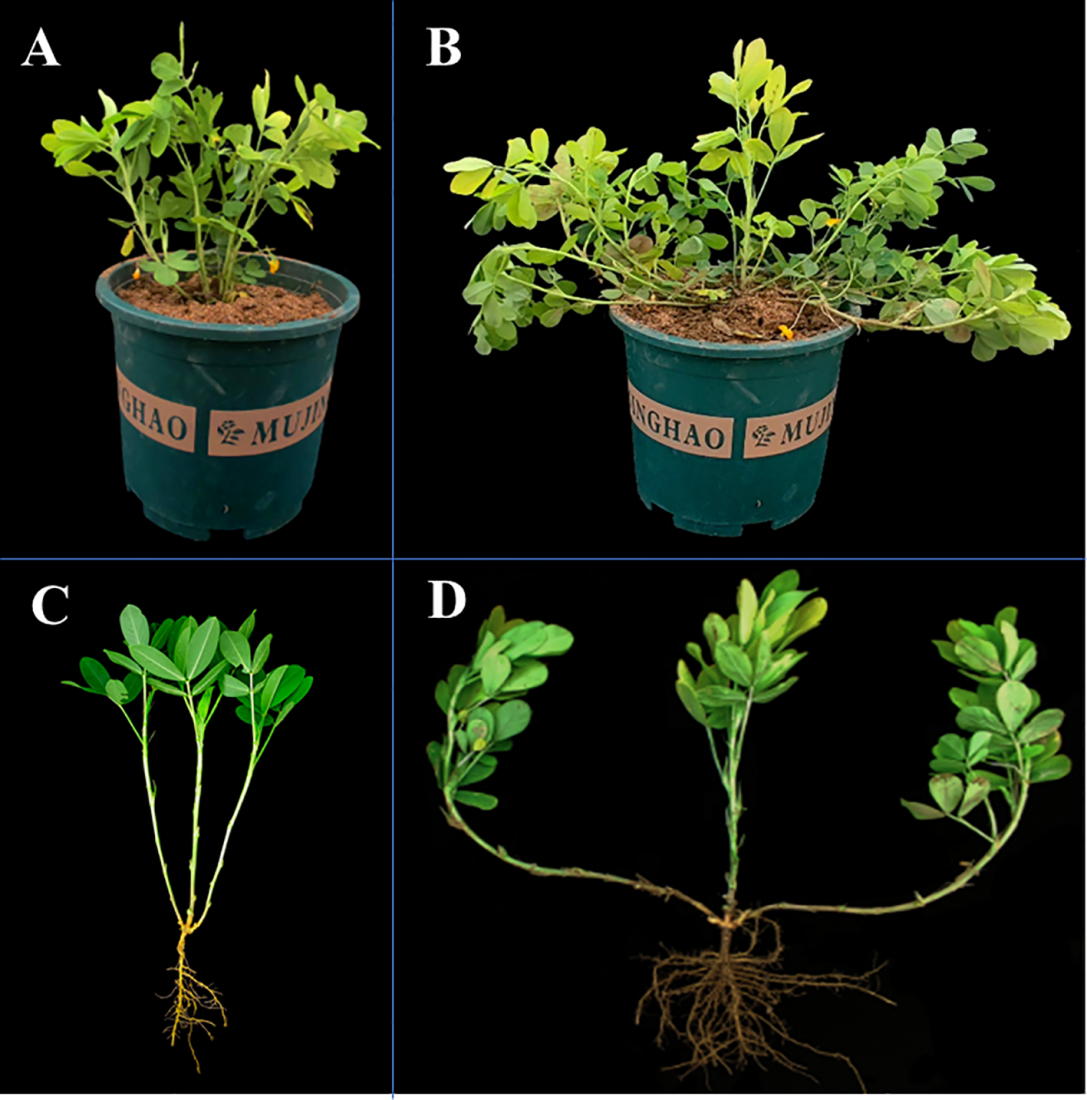
Different phenotypes of the parents of secondary populations. Jihua 5 erect growth **(A, C)**, PZ42 spreading-type growth **(B, D)**.

Our laboratory used the erect variety Jihua 5 and the prostrate variety M130 for hybridization to construct an RIL population. Dr. Li Li used parents and extreme offspring for BSA-seq, and the results showed that there were genes related to peanut lateral branch angle on chromosome B05 at 142610834-146688220 (4.08M, named *qGH*). Through screening in the RIL population, it was found that the genetic background of PZ42 is the same as that of the prostrate parent M130 in the *qGH* interval, and the lateral branch of PZ42 grow prostrate. So PZ42 was backcrossed with the erect parent Jihua 5 to construct a secondary population. Jihua 5 is a recurrent parent, while PZ42 is a non-recurrent parent. Remove the anthers of Ji Hua 5 the day before pollination. Teach the pollen of PZ42 to Jihua 5 during pollination (**Figures 1**, **2**). By backcrossing, recombination occurs within the interval of *qGH*, and the phenotype data of the recombinant plants are used to fine mapping *qGH*.

### Field experiment design and statistical analysis

In July 2021, Hebei Agricultural University West Campus build a secondary population. Backcross seeds were harvested in October. The BC1F1 population was planted in Sanya, Hainan Province (109.16E; 18.19N) in November. Seeds were harvested in March 2022, planted in May at Qingyuan Experimental Base of Hebei Agricultural University in Baoding, Hebei (115.56 E; 38.79 N)., and individual plants were harvested in October. and measure the angle between the two first lateral branches of each recombinant plant using an electronic digital protractor. The angle between the first pair of lateral branches and the main stem is half of the angle between the two first lateral branches. Then use Microsoft Excel for analysis and processing. When planting secondary population, the row spacing is 35 centimeters, the plant spacing is 10 centimeters, and 10 plants are planted per row. Cultivation techniques and field management measures are tailored according to local agricultural standards.

### QTL interval marker insertion, recombinant single plant screening

In June, collect leaves from individual plants for DNA extraction. After identifying InDel in the target region through genome resequencing of M130 and Jihua 5, obtain the reference genome sequence 200bp upstream and downstream of InDel from PeanutBase (https://peanutbase.org/). Design labeled primers using Primer 5.0 and synthesize them at Sangon Biotech. Extract DNA from the parents of the subpopulation using the CTAB method, set up the PCR reaction system, and conduct PCR. Electrophorese the PCR products on a 12% polyacrylamide gel for 2 hours. Identify polymorphic markers between the parents based on the banding pattern of the PCR products. Extract DNA from each individual plant in the subpopulation, perform PCR using polymorphic primer markers, run polyacrylamide gel electrophoresis for 2 hours, analyze the band type, and screen for recombinant individual plants within the *qGH* interval.

### RNA extraction and real-time

Jihua 5 and M130 varieties were planted in the field. When the first lateral branch reached lengths of 1 cm, 2 cm, 5 cm, and 10 cm, samples were taken from the junction of the peanut main stem and the first lateral branch tissue. These samples were rinsed with ddH2O, snap frozen in liquid nitrogen, and stored at -80 °. RNA extraction was carried out from the junction of the first lateral meristem and main stem tissues of both prostrate and bunch peanut varieties using the RNAprep Pure Plant Plus Kit (TIANGEN, China) according to the manufacturer’s instructions. Subsequently, 1 µg of RNA from each sample was analyzed on a 1% agarose gel for integrity and purity using a Thermo Scientific NanoDrop 2000. Gene sequences of *Arahy.C4FM6Y* were downloaded from the reference genomes available at https://dev.peanutbase.org/. Primers were designed based on the CDS region sequence of *Arahy.C4FM6Y* using Primer 5.0 and synthesized by Sangon Biotech. The cDNA was obtained by reverse transcription, and the real-time quantitative system was configured according to the instructions of the kit. The *ADT* gene (alcohol dehydrogenase class-3) of peanut was used as the internal reference (**Table 1**). Using LightCycler ^®^ 96 instrument was used for qPCR. First, pre denaturation at 95 ° for 2 minutes, followed by 40 cycles of denaturation at 95 ° for 5 seconds, renaturation at 58 ° for 10 seconds, and extension at 72 ° for 15 seconds. Using LightCycler ^®^ 96 SW 1.1 read qPCR results, 2^-△△Ct^ converted relative expression in the Microsoft Excel.

**Table 1:** Primer sequences for real-time quantification.

Gene	Forward primer	Reverse primer
*Arahy.C4FM6Y*	GGCGGGAATAAGGGTGCA	CCCAATTGCAAAGCCACC
*ADT*	GACGCTTGGCGAGATCAACA	AACCGGACAACCACCACATG

## Results

### Marker-encrypted of QTL interval

In the resequencing data of M130 and Jihua 5, a total of 233 indels were identified within the QTL interval. Subsequently, markers were developed based on the distribution of these indels. Through polyacrylamide gel electrophoresis, 10 markers were pinpointed to enhance the density of the QTL region. Notably, these 10 markers displayed substantial polymorphism among the parental lines of the secondary population (**Table 2**).

**Table 2:** Marker positions and upstream and downstream primer sequences of encrypted QTL.

Marker	Chr.	InDel Physical position	Forward primer	Reverse primer
MarInD1	15	143114773	CCATCATTCCTTGCCAAGAC	GCGCCATTTTGATGAAGGA
MarInD2	15	143485738	GGCTGACGGGAAGGCTC	CCCGTACCAACACCGCTG
MarInD3	15	143830120	GCTGCCTAATCACACTACAAGAGC	TGGGCCAATCTAGACCAAGTC
MarInD4	15	144313051	CGGCAAACCATGGTGCTT	CAGGATTCAAAACCCAGGCT
MarInD5	15	144926397	ACTGGCCCCTATGTGTCGTAC	GGATATCCATCTCTTGGAAACTAAC
MarInD6	15	145436466	GCTCTGGCGGCTACCTCA	GCGACGCGAGAAAGGAGG
MarInD7	15	145835004	AGCCGTGTCGTGACAGGG	TCTGAACCTCTGCTTTCCTGC
MarInD8	15	146070693	GCAATTTGAGAATATAGTGGGGC	AGAAAGATGCGGACGTCGAG
MarInD9	15	146269380	AGTCCAGCCTGTCGCGTC	GCAGGCTCACTCGCTCCA
MarInD10	15	146456746	CCTAACCTACTCTATCCATTGCC	GGCTATTGACACTTGACCTTTG

### Single plant screening and phenotype analysis

**Figure 3:**
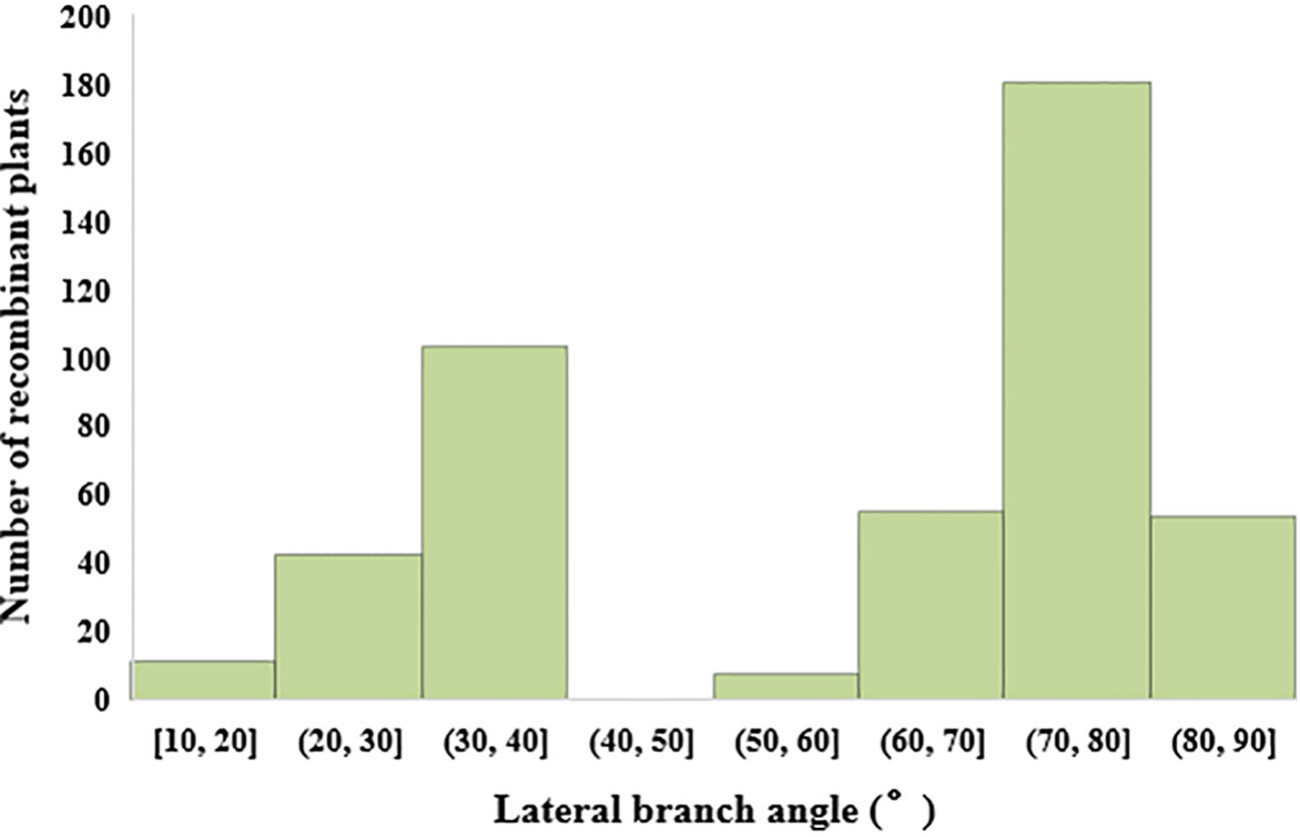
Histogram of lateral branch angle distribution of recombinant individual plants in secondary populations.

In the study on single plant selection and phenotypic analysis, 8000 individual plants were obtained from the BC_1_F_2_ secondary population. Selections were conducted using ten molecular markers, leading to the identification of 464 plants displaying recombination within the QTL interval. The lateral branch angles of the recombinant plants ranged from 10.00° to 90.00°, with an average angle of 58.67°, SS is 489.25. The lateral branch angles of the two parents are significantly different, with Jihua 5 having a lateral branch angle of 27.33° and PZ42 having a lateral branch angle of 85.39° (**Table 3**; **Figure 3**).

**Table 3:** Statistical Analysis of collateral angle of secondary population.

	Secondary Population				Parent	
	Average	SS	Min	Max	Jihua5	PZ42
LBA	58.67	489.25	15.00	90.00	27.33	85.49

### Fine-mapping of lateral branch angles

**Figure 4:**
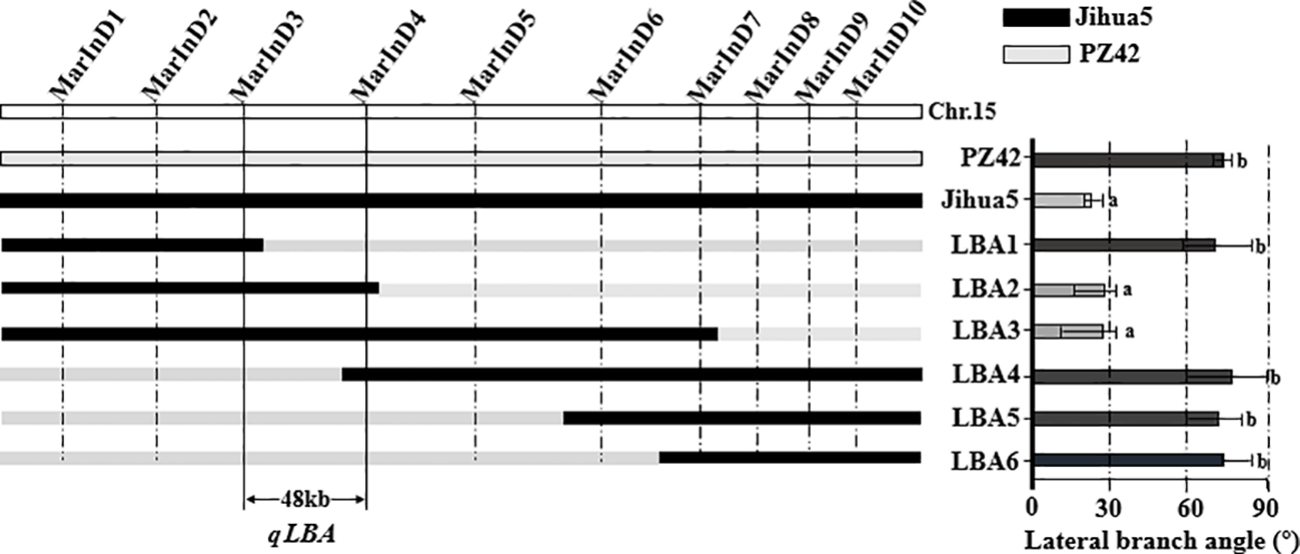
Fine positioning analysis of peanut lateral branches. Different letters (a: High significance; b: significance) indicate significant at P ≤ 0.05 (Tukey-HSD).

In this experiment, phenotypic data from the BC_1_F_2_ secondary population and inserted molecular markers were analyzed using one-way ANOVA to identify markers closely related to the lateral branch angle of peanuts. The aim was to narrow down the QTL interval for fine mapping. Molecular markers were used to identify recombinant plants with heterozygous regions that covered the entire QTL interval. The results of one-way ANOVA between the lateral branch angle and each marker revealed a high correlation between the band patterns of MarInD3 and MarInD4 markers and the size of the lateral branch angle. After analyzing phenotypic and genotype data from the BC_1_F_2_ secondary population, results from target segment 1 showed that six genotypes (LBA 1-6) spanned the entire QTL interval (**Table 4**). These six genotypes had lateral branch angles of 59.79°-86.27°, 15.14°-33.96°, 10.00°-33.64°, 60.00°-90.00°, 60.37°-81.00°, and 65.34°-84.29°, respectively, with significant differences observed. According to phenotype analysis, the phenotypes of LBA2 and LBA3 are similar to those of Jihua 5, and the lateral branch angle of peanut is significantly smaller than other genotypes. After comparing various genotypes, it was found that when the MarInD3-MarInD4 interval is contributed by Jihua 5, the angle of peanut lateral branches is small, and the first pair of lateral branches of the recombinant individual plant of this genotype will grow erect; When the MarInD3-MarInD4 interval is contributed by PZ42, the lateral branch angle of peanut is large, and the first pair of lateral branches of this genotype of recombinant individual plant will grow prostrate. (**Table 5**). The comprehensive results indicate that the presence of genes related to peanut lateral branch angle between markers MarInD3-MarInD4. Following fine mapping, the QTL interval was reduced from 4.08 Mb to 48 kb, and the new interval was named *qLBA* (**Figure 4**).

**Table 4:** ANOVA between lateral branch angles and markers of recombinant individual plants.

		SS	Df	MS	F	Significance (P>0.05)
MarInD3	Interblock	121677.90	1	121677.90	537.76	Highly Significant
	Group	104760.77	463	226.27		
	Σ	226438.66	464			
MarInD4	Interblock	85950.43	1	85950.43	283.26	Highly Significant
	Group	140488.24	463	303.43		
	Σ	226438.66	464			

**Table 5:** Number of recombinant plants were identified for each group.

Genotype	Lateral branch angle	Number of recombinant individual plants
LBA 1	59.79°-86.27°	59
LBA 2	15.14°-33.96°	140
LBA 3	10.00°-33.64°	20
LBA 4	60.00°-90.00°	98
LBA 5	60.37°-81.00°	67
LBA 6	65.34°-84.29°	80

### Screening of candidate genes

Retrieve genes from *qLBA* in PeanutBase based on the physical location of the marker. A search found 14 genes within this interval (**Table 6**). Further examination of gene annotations revealed that *Arahy.B5N2MW*, *Arahy.EID17C*, *Arahy.8H2QTH*, *Arahy.RU949X* and *Arahy.J45ZPD* translates unknown proteins, *Arahy.XU3UAF* and *Arahy.8PB8XJ* participate in glycine metabolism and protein synthesis, *Arahy.G37EV9*, *Arahy.K6T9FH*, and *Arahy.02FBPF* participate in ATP synthesis metabolism, *Arahy.122XK3* controls the synthesis of retinal family protein, *Arahy.FR68MS* controls the synthesis of histidyl tRNA synthetase 1, and *Arahy.PL71TD* translates UDP-Lycosyltransferase superfamily protein, *Arahy.C4FM6Y* encodes an F-box protein. Naveed’s study showed that peanut collateral growth was affected by gravity sensing mechanism, which affected collateral angle by stimulating starch and sucrose metabolism as well as Cytokinins (CKs), Strigolactones (SLs) and auxin signals. In this metabolic pathway, F-box protein will inhibit the conversion of CKs into HSF, affect the content of CKs at the base of peanut lateral branches, and then affect the angle of peanut lateral branches. Among the 14 genes, only *Arahy.C4FM6Y* is involved in regulating peanut lateral branch angle, while other genes are not directly or indirectly involved in regulating peanut lateral branch angle. As a result, *Arahy.C4FM6Y* has been identified as a potential candidate gene that influences the angle of lateral branch, and rename *Arahy.C4FM6Y* as *Aralbab05*.

**Table 6:** Genes within *qLBA* and their annotations.

Gene	Chr	Position	Length	Description
*Arahy.B5N2MW*	15	143957498-143959375	1578	Unknown protein
*Arahy.8H2QTH*	15	143927832-143932815	4984	Unknown protein
*Arahy.EID17C*	15	144077724-144079580	1857	Unknown protein
*Arahy.RU949X*	15	144198636-144199391	756	Unknown protein
*Arahy.XU3UAF*	15	144088145-144089593	1449	serine/threonine-protein phosphatase 7 long form homolog [Glycine max]; IPR019557 (Aminotransferase-like, plant mobile domain)
*Arahy.J45ZPD*	15	144091979-144094574	2596	uncharacterized protein LOC100793641 isoform X1 [Glycine max]
*Arahy.122XK3*	15	144098769-144101094	2326	Reticulon family protein; IPR003388 (Reticulon)
*Arahy.FR68MS*	15	144144269-144146671	2403	Histidyl-tRNA synthetase 1; IPR018609 (Bud13)
*Arahy.G37EV9*	15	144150249-144161759	11511	ATP binding microtubule motor family protein; IPR001752 (Kinesin, motor domain), IPR002885 (Pentatricopeptide repeat), IPR006458 (Ovate protein family, C-terminal), IPR011990 (Tetratricopeptide-like helical), IPR027417 (P-loop containing nucleoside triphosphate hydrolase); GO:0003777 (microtubule motor activity), GO:0005515 (protein binding), GO:0005524 (ATP binding), GO:0007018 (microtubule-based movement), GO:0008017 (microtubule binding)
*Arahy.8PB8XJ*	15	144175851-144179769	2973	Pentatricopeptide repeat (PPR) superfamily protein; IPR002885 (Pentatricopeptide repeat), IPR011990 (Tetratricopeptide-like helical); GO:0005515 (protein binding)
*Arahy.C4FM6Y*	15	144202961-144203966	1006	F-box plant-like protein, putative; IPR027949 (Petal formation-expressed)
*Arahy.K6T9FH*	15	144257376-144264510	7135	ATP-binding microtubule motor family protein; IPR001752 (Kinesin, motor domain), IPR021881 (Protein of unknown function DUF3490), IPR027417 (P-loop containing nucleoside triphosphate hydrolase), IPR027640 (Kinesin-like protein); GO:0003777 (microtubule motor activity), GO:0005524 (ATP binding), GO:0005871 (kinesin complex), GO:0007018 (microtubule-based movement), GO:0008017 (microtubule binding)
*Arahy.02FBPF*	15	144295446-144299612	4167	ATP-binding microtubule motor family protein; IPR001752 (Kinesin, motor domain), IPR027417 (P-loop containing nucleoside triphosphate hydrolase), IPR027640 (Kinesin-like protein); GO:0003777 (microtubule motor activity), GO:0005524 (ATP binding), GO:0005871 (kinesin complex), GO:0007018 (microtubule-based movement), GO:0008017 (microtubule binding)
*Arahy.PL71TD*	15	144301609-144302572	964	UDP-Glycosyltransferase superfamily protein; IPR002213 (UDP-glucuronosyl/UDP-glucosyltransferase); GO:0008152 (metabolic process)

### Real-time quantification of *Aralbab05*

**Figure 5:**
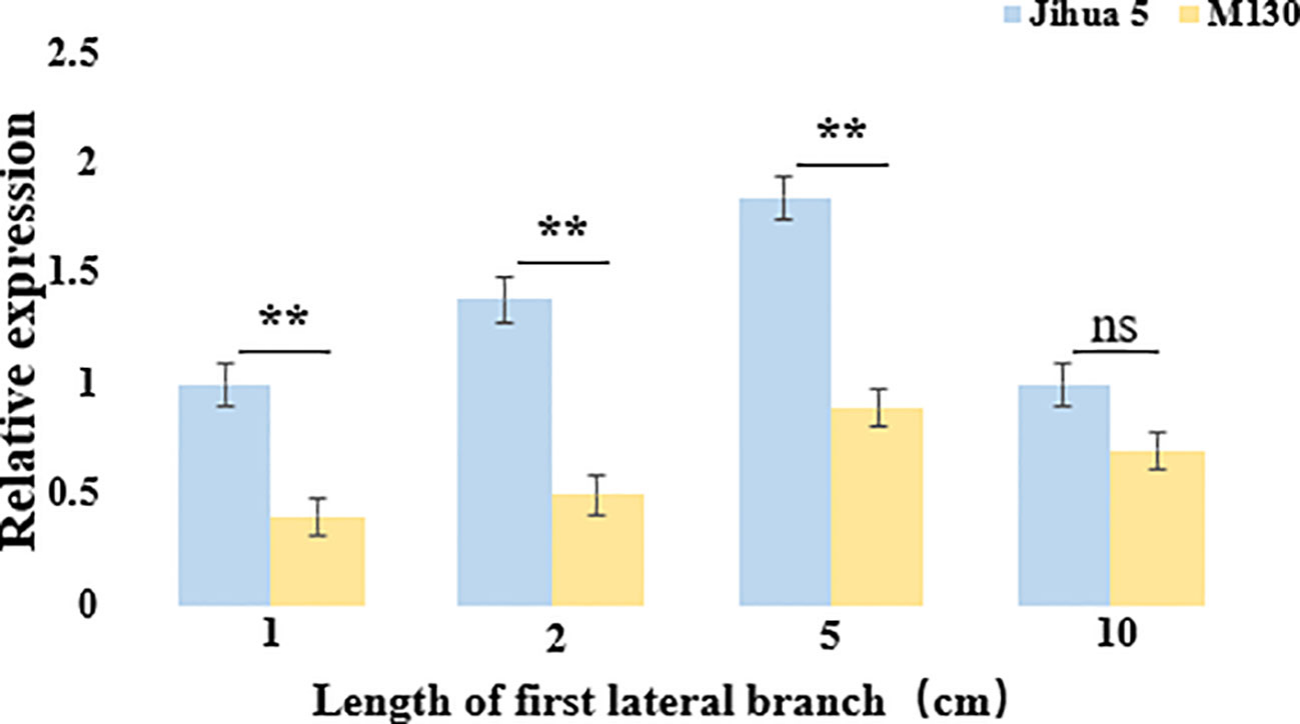
Expression of *Arahy.C4FM6Y* at the junction between the main stem and the first lateral branch of Jihua 5 and M130. Each data set represents three biological replicates, ns means no statistical significance, and an asterisk means statistically significant (one-way ANOVA; **, P<0.01).

To verify whether *Aralbab05* has an effect on the angle of peanut lateral branches during their development, the *Aralbab05* gene expression was analyzed. The expression level of *Aralbab05* in Jihua 5 and M130 during the development of the first lateral branch of peanuts shows a pattern of initial increase followed by a decrease. Specifically, as the lateral branch grows from 1 cm to 5 cm, the expression of *Aralbab05* rises, peaking at 5 cm. Subsequently, from 5 cm to 10 cm, there is a decline in *Aralbab05* expression. Notably, during the development of the first lateral branch of peanut, the relative expression of *Aralbab05* in Jihua 5 was higher than that in M130, particularly significantly at 2 cm and 5 cm lengths, The relative expression of *Aralbab0*5 in Jihua 5 and M130 at these two time points was significantly different, which also showed that these two time points were the critical period for the formation of peanut lateral branch angle. These findings indicate that *Aralbab05* plays a role in inhibiting the increase of lateral branch angle during the first lateral branch development in peanuts, aligning with the real-time quantitative outcomes. It is suggested that *Aralbab05* may play a more important role in the formation of peanut lateral branch angle. (**Figure 5**).

## Discussions

Multiple studies have shown that the angle of branching is determined by the uneven distribution of auxin, which is influenced by gravity and light. This distribution is affected by auxin synthesis, transport, and signal transduction, which in turn lead to asymmetric growth induced by auxin. When it comes to gravity sensing, the amyloplasts in stems can sense gravitational cues and move accordingly, playing a pivotal role in this process (Sack, 1997). Additionally, research on rice stems has revealed that the *AGPL1* and *agpl1agpl3* double mutants inhibit starch synthesis, resulting in a decreased gravitropic response and an increased tiller angle (Okamura et al., 2013, 2014). Furthermore, genes such as *SGR*, *SGR2*, *SGR3*, *SGR4*, *SGR5*, *SGR6*, and *SGR9* in Arabidopsis are involved in regulating the deposition of amyloplasts, thereby influencing the gravity response (Hashiguchi et al., 2013; Nakamura et al., 2011; Tanimoto et al., 2008). In rice, the *LPA1* gene, which is a counterpart to the *SGR5* gene, controls the sedimentation rate of amyloplasts to impact the plant’s gravitropic response. Moreover, in collaboration with the transcription factor *ONAC106*, it also affects the tiller angle (Morita et al., 2006; Wu et al., 2013; Sakuraba et al., 2015).

Studies have shown that Strigolactone (SL) can decrease the tiller angle by inhibiting auxin biosynthesis (Sang et al., 2014). The *Prog1* (*Promote Growth 1*) gene is responsible for promoting ground growth in rice, with mutations causing the plant to grow upright (Jin et al., 2008; Tan et al., 2008; Hu et al., 2018). Research on rice has found that *LAZY1* and *LAZY4* regulate the gravity and tiller angle of branches, leading to lateral auxin transport under gravity stimulation and changes in tiller angle caused by uneven redistribution of auxin (Wang et al., 2024; Li et al., 2019b). African cultivated rice and Asian cultivated rice exhibit creeping and erect growth types, respectively, with deletions of approximately 110 Kb and 113 Kb in their prog1 gene. Additionally, within this region, there are seven tandem zinc finger protein genes that may play a role in regulating tiller angle (Wu et al., 2018).

The research indicates that plants exhibit phototropic growth, with branches bending towards light due to the uneven distribution of auxin. Similar to gravitropism, phototropism involves light signal perception, transduction, auxin distribution, and organ curvature. Throughout plant development, a shade avoidance response ensures that plant branches and leaves grow upwards to maximize sunlight exposure (Roychoudhry et al., 2017; Ballaré et al., 1990). Research has demonstrated that in shaded conditions, such as with corn, phytochrome-mediated signals trigger an increase in tb1 expression, leading to branch inhibition. This response is also observed in sorghum and Arabidopsis (Kebrom et al., 2006; Whipple et al., 2011; Yasuko et al., 2014; Xie et al., 2020).

Waite demonstrated that the *TAC1* (*Tiller Angle Control 1*) gene is light-dependent and responsive to light signals for regulating lateral branch angles (Waite and Dardick, 2018). In dark conditions, the core inhibitor of the light signal transduction pathway, COP1, inhibits *TAC1* expression in Arabidopsis thaliana, leading to reduced branch angles. Studies in rice by have shown that higher *TAC1* expression correlates with larger tiller angles (Li et al., 2007). Similarly, in maize, mutations in the *TAC1* gene result in smaller leaf angles and a more compact plant structure, as described by (Ku et al., 2011). These findings indicate a conserved role of *TAC1* in controlling branch and leaf angles in different plant species, including Arabidopsis.

**Figure 6:**
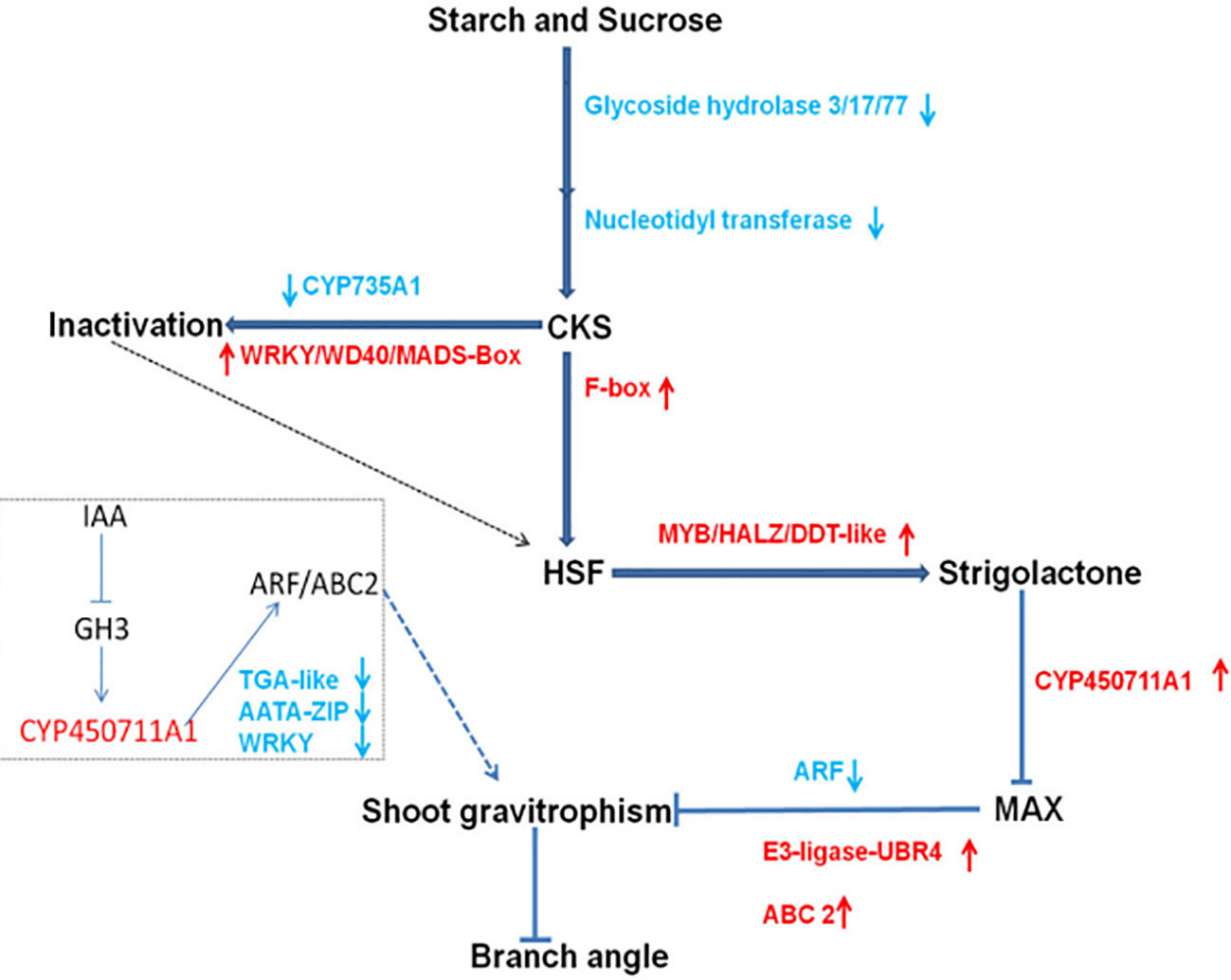
The proposed regulatory network underlying branch angle formation in peanut.

In the research of peanut plant morphology three genes related to hormone metabolism were identified: one encoding ‘F-box protein’, another encoding 2-oxoglutarate, and the third encoding ‘Fe(II)-dependent oxygenase’. The research also revealed notable variations in cytokinin, auxin, and ethylene levels at the junction of the main stem and lateral branches between prostrate and upright peanuts (Pan et al., 2022). Through transcriptome data analysis, Ahmad identified 13 genes associated with gravity response, 22 genes linked to plant hormones, and 55 transcription factors. Additionally, he conducted a preliminary analysis of the metabolic pathways related to peanut lateral branch angles (**Figure 6**) (Ahmad et al., 2022).

Research on the lateral branch angle of peanuts is currently less advanced compared to crops like rice and wheat. To develop high-yielding peanut varieties suitable for mechanized harvesting, it is crucial to delve deeper into gene mechanisms, enhance metabolic pathway regulation, and accelerate the breeding process. Additionally, the development of specialized machinery for peanuts can help reduce labor costs and enhance overall efficiency.

## Conclusion

In this study, the secondary population constructed by the hybrid progeny PZ42 and Jihua 5 was used to fine map the lateral branch angle of peanut, and the QTL interval length was reduced from 4.08Mb to 48kb. The genes in the fine-mapping interval were screened, that the *Aralbab05* gene encoding F-BOX protein was identified as a candidate gene related to the lateral branch angle of peanut, which was quantitatively verified in Jihua 5 and M130 in real-time. The real-time quantitative results showed that when the lateral branches of peanut were 1-10 cm, the expression of *Aralbab05* in Jihua 5 was higher than that in M130.

## Data Availability

The datasets presented in this study can be found in online repositories. The names of the repository/repositories and accession number(s) can be found in the article/supplementary material.
